# Prevalence, risk perception, and motivations behind E-cigarettes and heated tobacco use: a cross-sectional study in Italian adolescents

**DOI:** 10.1186/s13052-025-02144-y

**Published:** 2025-11-17

**Authors:** Fabrizio Virgili, Fabiola Del Parco, Domenico Paolo La Regina, Enrica Mancino, Laura Petrarca, Maria Giulia Conti, Enea Bonci, Raffaella Nenna, Fabio Midulla

**Affiliations:** Department of Maternal Infantile and Urological Sciences, Sapienza University of Rome, Viale Regina Elena 324 - 00161 , Rome, Italy

**Keywords:** Adolescents, E-Cigarettes, Heated tobacco products, Youth, Vaping

## Abstract

**Background:**

E-cigarettes and Heated Tobacco Products (HTPs), which emerged as “safer” alternatives to traditional cigarettes, remain easily accessible and widely misperceived as harmless, especially among adolescents. Our study aimed to assess the prevalence of e-cigarette and HTP use among Italian adolescents, and investigate underlying motivations, risk perceptions, and social influences.

**Methods:**

We conducted a cross-sectional survey among 200 adolescents aged 11–18 years. Participants completed a 50-item anonymous questionnaire exploring sociodemographic characteristics, smoking behaviors, reasons for use, risk awareness, and social influence.

**Results:**

Among participants, 23% reported using e-cigarettes and 16% HTPs. Disposable, flavored and nicotine-containing products were highly prevalent. Among vapers, use was significantly more frequent in those aged > 14 years, and in individuals reporting social influence, stress, or sadness/apathy. Similar patterns were observed among HTP users. Risk perception was low: 85% of participants believed e-smoking was less harmful than conventional smoking, and only 5% recognized e-cigarettes as significantly harmful. Only 20% of all participants had been asked about smoking during medical visits, with significantly lower rates among those < 14 years. A comprehensive analysis of usage patterns, psychosocial correlates, and risk perception is provided in the full manuscript.

**Conclusion:**

The underestimation of health risks associated with electronic smoking devices pose a serious Public Health challenge. Findings highlight the urgent need for targeted interventions combining stricter access control, enhanced risk communication, and integration of tobacco prevention into school and clinical settings. Greater attention should be paid to the psychological dimension of adolescent smoking and the evolving landscape of nicotine delivery systems.

**Supplementary Information:**

The online version contains supplementary material available at 10.1186/s13052-025-02144-y.

## Background

Smoking constitutes the world’s leading cause of preventable diseases, contributing to millions of premature deaths and to a significant burden of chronic diseases, including cancer, cardiovascular and respiratory diseases, therefore exerting a devastating impact on healthcare systems. In the last twenty years, marketing restrictions, prevention campaigns and increased taxation contributed to a slow - albeit progressive - decline in smoking prevalence [1, 2]. 

Despite such efforts, the landscape of nicotine consumption has been reshaped by the introduction of e-cigarettes (e-cigarettes or E-cigs) and Heated Tobacco Products (HTPs). E-cigarettes are electronic devices consisting of a battery, an electric heater and an aerosolized liquid for inhalation [3, 4]. Although marketed (and therefore perceived) as a less harmful alternative to traditional smoking, vaping poses significant health risks, especially with regard to respiratory and cardiovascular health, addictive potential, and mental well-being (impaired neurodevelopment, cognitive deficits and mood disorders), with adolescents being particularly vulnerable [5–9]. Heated tobacco products heat reconstituted tobacco to temperatures between 350 and 550 °C, producing an aerosol which contains lower concentrations of tobacco-specific nitrosamines, carbon monoxide and volatile organic compounds than conventional cigarettes, but with comparable nicotine emissions and sometimes higher levels of reactive oxygen species and pyrolysis products [10, 11]. Accumulating evidence suggests that also HTPs can produce adverse pulmonary, cardiovascular and immunomodulatory effects [10, 12–14]. 

In contrast to the progressive decline in youth tobacco smoking, e-cigarettes have rapidly become the most popular tobacco product among adolescents. This was largely due to the general misconception of their safety, reinforced by marketing portraying them as modern, harmless nicotine alternatives. Social acceptance and easy access have significantly increased both adolescent use and children’s second-hand exposure, emphasizing the need for an assessment of their health impact in such populations [4, 15, 16]. Similarly, the tobacco industry’s harm reduction narrative surrounding HTPs emphasized reduced exposure to harmful constituents, yet overlooked the broader Public Health impact, including the risk of reduced harm misperception and the potential influence on youth initiation [17]. Weak age verification systems, both in-store and online, further facilitate youth access. Aggressive marketing campaigns, in a scenery where smoking is both easily accessible and socially accepted, has led to a real epidemic among adolescents, a concerning trend demanding stricter regulatory interventions and more rigorous enforcement measures [18–20]. 

The rise of e-cigarettes use among adolescents concerned also Europe, with 10.6% reporting ever-use and 6.0% currently using e-cigarettes [21]. Current e-cigarette’s use among children aged 13–15 years frequently exceeds that observed in older groups, with higher prevalence in boys than girls in most countries. Alarmingly, several European countries now report e-cigarette’s use among adolescents surpassing cigarette smoking, in some cases by two- to three-fold [22]. In Italy, the prevalence of adolescent smokers (including both traditional and e-cigarettes) aged 13–16 increased from 20.7% in 2010 to 27.9% in 2018. Over the same period, current e-cigarette use rose sharply from 0% in 2010 to 7.4% in 2014, reaching 17.5% in 2018, with the proportion of exclusive vapers tripling [23, 24]. The most recent data show that overall use of tobacco and nicotine products among adolescents remains high (30.2%), despite a declining trend for traditional cigarettes (20%) and e-cigarettes (18.5%). In stark contrast, HTPs have gained significant popularity, rising by 6.4% to reach 18.7%. This shift is particularly evident among younger adolescents, with experimentation rates increasing from 12% at age 14 to 23% by age 16 [25]. 

Smoking behavior results from a complex interaction of individual, psychosocial, and environmental factors, influencing both initiation and maintenance of tobacco use. Risk awareness plays a pivotal role, as individuals with limited knowledge of smoking-related health risks tend to underestimate its negative consequences, increasing their likelihood of smoking. Psychosocial influences further shape smoking behavior, with peer pressure and exposure to smoking role models within social and familial circles normalizing tobacco use [26, 27]. 

Our study aims not only to assess prevalence, but also to systematically integrate an analysis of underlying motivations, risk perceptions, and social influence surrounding electronic smoking among Italian adolescents, factors being often investigated independently or with limited scope. While previous research has predominantly focused on e-cigarettes, our study uniquely addresses the emerging role of HTPs in youth nicotine consumption. This comprehensive approach allows for a deeper understanding of the behavioral determinants influencing their uptake and provides a solid basis for targeted Public Health interventions.

## Materials and methods

### Objectives

The primary objectives of the present study are: (1) to assess the prevalence of electronic cigarette and heated tobacco products’ use among young individuals; (2) to analyze youth perceptions and motivations regarding electronic smoking devices. By investigating these aspects, our goal is to provide insights into the determinants of smoking behavior in younger populations in order to define targeted preventive strategies.

### Study design

This is an epidemiological observational study, carried out at the Sapienza University in Rome between December 2024 and March 2025. Data collection was conducted through a structured online survey created using Google^®^ Forms platform. The survey was derived from a reduced version of the Global Youth Tobacco Survey, adapted to the Italian epidemiological context with a specific focus on e-cigarettes and heated tobacco products [28]. The reduction aimed to balance comprehensiveness with feasibility of administration in an outpatient setting. It consisted of 50 multiple-choice questions specifically designed to gather detailed information on smoking habits and underlying motivations. It was divided into sections covering the following domains: age, gender, and education level; nature, frequency, and intensity of smoking habits; reasons for smoking; risk perception; family and social influences. The full version of the survey is available in supplementary material. The questionnaire was administered digitally via QR code scanning to a sample of 200 adolescents aged 11–18 years, equally distributed by gender. Participation was voluntary, and no incentives were provided to the respondents. Exclusion criteria included the absence of informed consent and the presence of severe chronic diseases, including respiratory conditions (i.e. asthma). Participants completed the survey anonymously and independently, which ensured that answers were not influenced by parental supervision. Subjects were recruited after obtaining written informed consent from parents or legal guardians.

### Statistical analysis

The collected data were initially processed using the automatic response aggregation function in Google^®^ Forms. Subsequently, qualitative and quantitative variables were organized in Excel spreadsheets and imported into an electronic database for statistical analysis. Descriptive statistics were used to summarize the characteristics of the study population. Categorical variables were analyzed using the chi-square test. A p-value < 0.05 was considered statistically significant. Statistical analyses were performed using IBM SPSS Statistics.

## Results

A total of 200 participants were recruited, 105 (53%) males. The age distribution was as follows: 11 years 9%, 12 years 10%, 13 years 11%, 14 years 12%, 15 years 13%, 16 years 14%, 17 years 15%, and 18 years 16%.

**Fig. 1:**
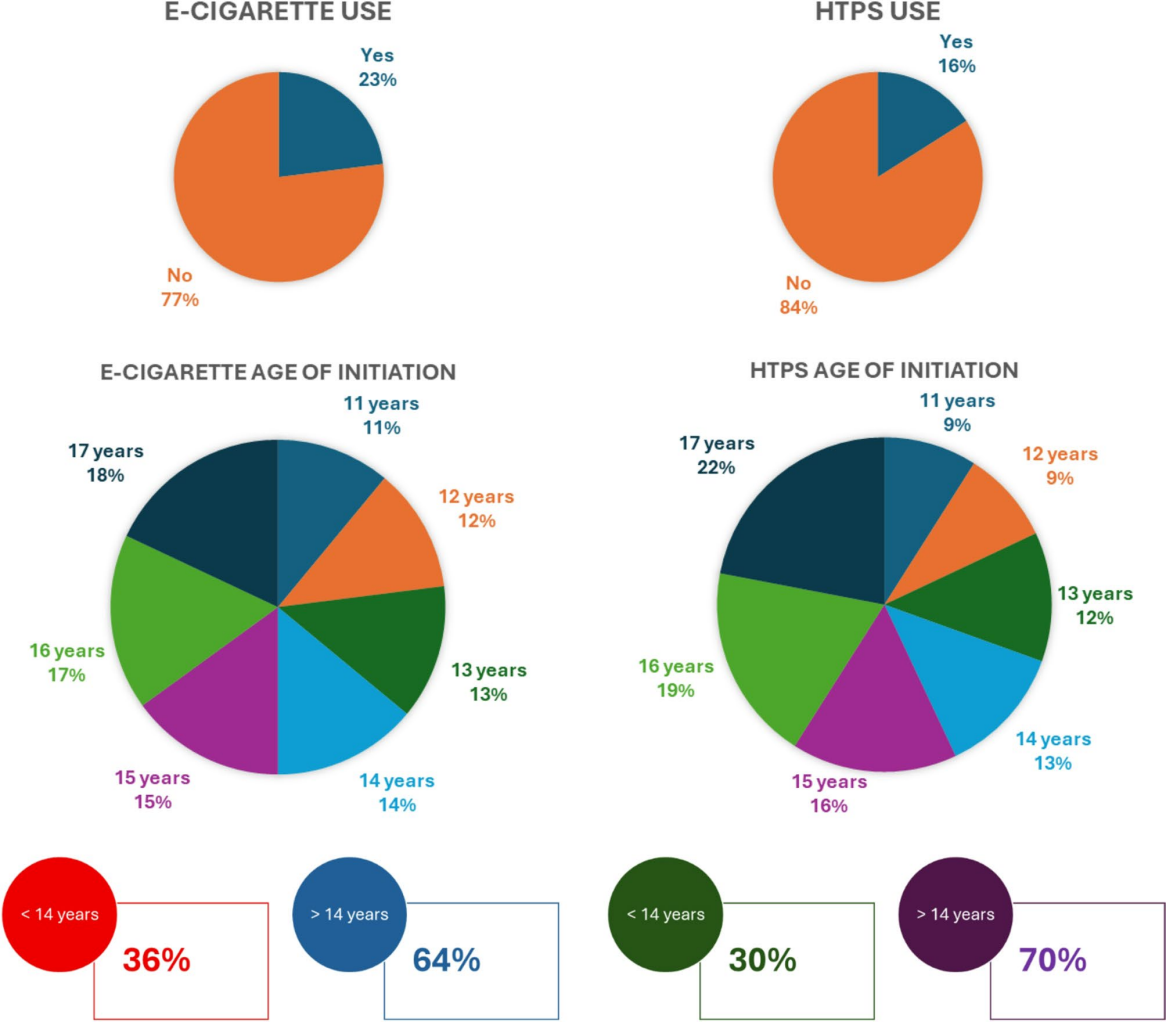
E-cigarettes and Heated Tobacco Products prevalence and age of initiation

**Fig. 2:**
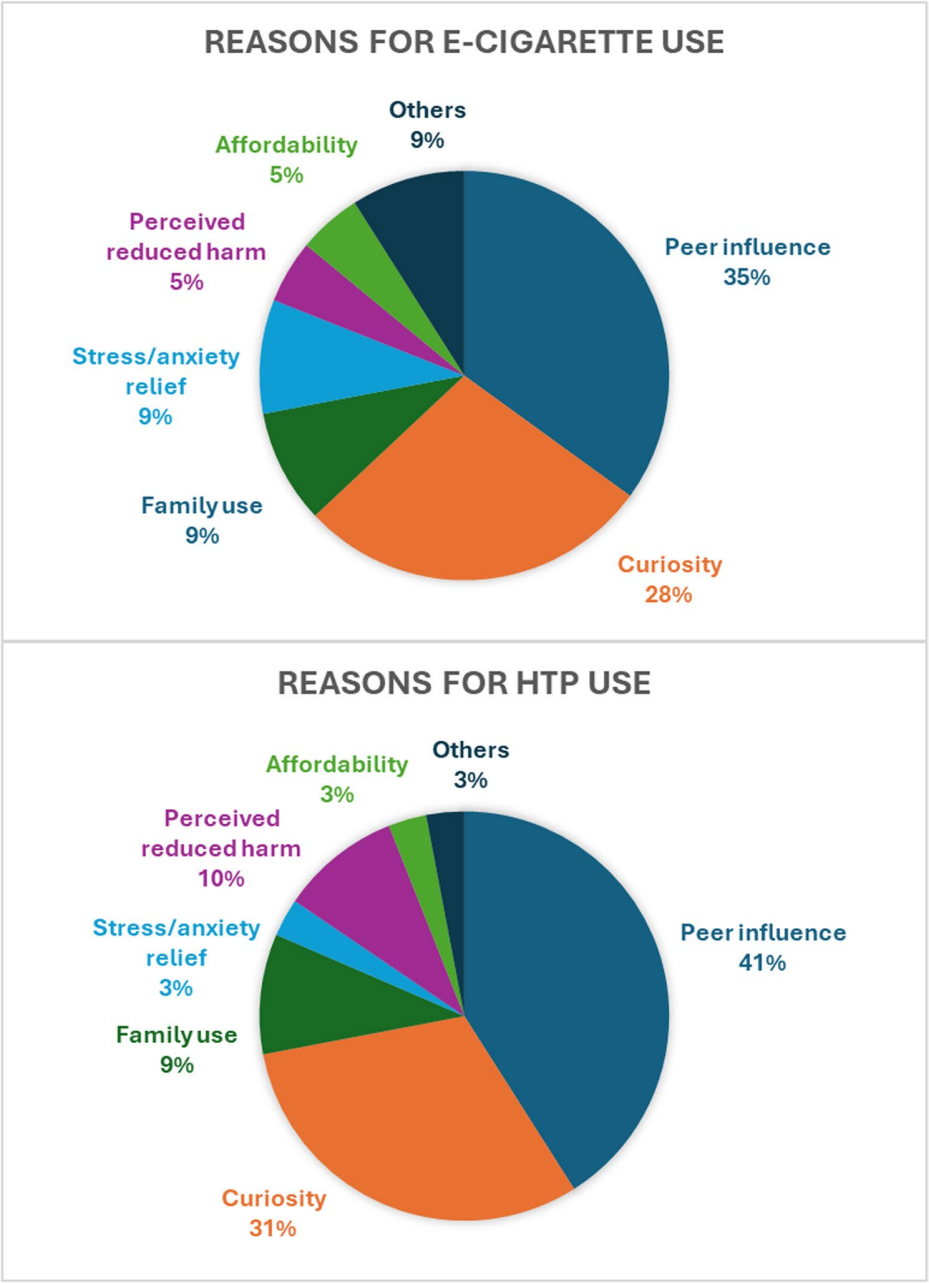
E-cigarettes and Heated Tobacco Products main reasons of use

The findings related to e-cigarette and/or heated tobacco products’ use, derived from the analysis of questionnaire responses, are presented in Figs. 1 and 2 and in Tables 1, 2, 3, 4, 5 and 6.

**Table 1:** Epidemiological data related to e-cigarettes’ use

Prevalence data	
* Ever-users*	46 (23%)
* Frequency among users*	1–2 times/month: 49% 3–9 times/month: 11% ≥ 10 times/month: 40%
* Device type used*	Disposable: 69% Refillable: 15% Pod-based: 19% Unsure: 7%
* Nicotine-containing e-cigarettes (last 30 days)*	91% (81% of users aged < 14 y.o. and 97% of users aged > 14 y.o.; *p* < 0.001)
* Flavored e-cigarettes*	76%
* Preferred flavors*	Fruit: 41% Menthol: 15% Sweet: 11%
Source/setting of acquisition	
* Personally purchased e-cigs*	52%
* Purchase sources*	Tobacconists: 28% Other individuals: 15% Vending machines: 9%
* Third-party providers of legal age*	40%
* Acquired at school*	22%
* Used someone else’s e-cigarette*	73% (Occasionally: 69%, Regularly: 4%)
Non-users intentions	
* Would probably try in future*	23%
* Would definitely try*	15%
* Would accept if offered by a friend*	Probably: 12% Definitely: 6%
Cessation Attempts	
* Never tried to quit*	30%
* Tried to quit*	70%
Quit attempts (among those who tried)	1–2 times: 37% 3–9 times: 39% > 10 times: 24%
* Sought support when quitting*	30%
* Support sources*	Friends: 3% Teachers/coaches: 3% Parents: 6% Others: 18%

**Table 2:** Epidemiological data related to heated tobacco products’ use

Prevalence data	
* Ever-users*	16%
* Male users*	47.2%
* Frequency*	1–2 times/month: 53% 3–9 times: 13% ≥ 10 times: 34%
* Nicotine-containing HTPs (last 30 days)*	94% (93% of users aged < 14 y.o. and 94% of users aged > 14 y.o.; *p* > 0.05)
* Flavored HTPs*	64%
* Preferred flavors*	Menthol: 61% Fruit: 29%
Source/setting of acquisition	
* Purchase sources*	Tobacconists: 34% Vending machines: 16% Third parties: 13%
* Third-party providers of legal age*	56%
* Minors purchasing directly*	4%
* Acquired at school*	28%
* Used someone else’s HTP*	56% (Occasionally: 53%, Regularly: 3%)
Non-user intentions	
* Would probably try in future*	60%
* Would definitely try*	16%
* Would accept if offered by a friend*	Probably: 15% Definitely: 5%
Cessation Attempts	
* Never tried to quit*	26%
* Tried to quit*	74%
* Quit attempts (among those who tried)*	1–2 times: 35% 3–9 times: 47% > 10 times: 18%
* Sought support when quitting*	30%
* Support sources*	Friends: 6% Teachers/coaches: 3% Parents: 3% Others: 18%

**Table 3:** Data related to participants’ risk awareness and perceptions

Health impact-related questions	
* Awareness of all tobacco products’ health risks*	61%
* E-cigarette’s health impact perception*	Significant harm: 5% Minimal: 51% No harm: 44%
* HTPs’ health impact perception*	Significant harm: 2% Minimal: 51% No harm: 47%
* E-smoking vs. traditional cigarettes*	Less harmful: 85% Same: 12% More harmful: 1% Don’t know: 2%
Do all e-cigs contain nicotine?	
* Yes*	53%
* Some*	39%
* No*	8%
Are e-cigs as addictive as cigarettes?	
* Equally addictive*	39%
* Less addictive*	13%
* Don’t know*	25%
Do all HTPs contain nicotine?	
* Yes*	35%
* Some*	52%
* No*	13%
Are HTPs as addictive as cigarettes?	
* Equally addictive*	34%
* Less addictive*	51%
* Don’t know*	15%

**Table 4:** Data related to participants’ secondhand exposure

Outdoor exposure frequency	
* Regularly*	16%
* 1–5 days/month*	49%
* 6–10 days*	13%
* 10–20 days*	15%
* 20–30 days*	7%
Indoor exposure frequency	
* Regularly*	11%
* 1–5 days/month*	32%
* 6–10 days*	12%
* 10–20 days*	11%
* 20–30 days*	4%
Household exposure	
* Regularly*	11%
* 1–5 days/month*	32%
* 6–10 days*	12%
* 10–20 days*	11%
* 20–30 days*	4%

**Table 5:** Participants’ emotional state

Emotional Self-Description	
* Calm*	29%
* Happy*	22.5%
* Apathetic*	16.5%
* Sad/Depressed*	20%
* Anxious*	12%

**Table 6:** Data related to prevention and healthcare

*Ever asked about smoking during medical visits*	20% (8% of children < 14 y.o. and 27% of children > 14 y.o; ***p* < 0.001**)
*Ever seen/heard anti-smoking campaigns*	52%

The comparison of the different variables associated with the use of e-cigarettes and heated tobacco products between smokers and non-users groups is presented in Tables 7 and 8, respectively.

**Table 7:** Comparison of variables associated with e-cigarettes’ use

Variables	E-cigarette Use No (%)	E-cigarette Use Yes (%)	Total (%)	*p* value
*Gender (Male)*	82/153 (53.6%)	23/47 (48.9%)	105/200 (52.5%)	0.576
*Age > 14 years*	86/153 (56.2%)	42/47 (89.4%)	128/200 (64%)	**< 0.001**
*Influenced by peers or family*	1/153 (0.7%)	22/47 (46.8%)	23/200 (11.5%)	**< 0.001**
*Stress*	0/153 (0%)	8/47 (17%)	8/200 (4%)	**< 0.001**
*Sadness/apathy*	52/153 (34%)	21/47 (44.7%)	73/200 (37%)	**< 0.001**
*Relatives smoke*	78/153 (51%)	36/47 (76.6%)	86/200 (43%)	**0.002**
*Belief that electronic smoking is less harmful than traditional cigarettes*	130/153 (85%)	40/47 (85.1%)	170/200 (85%)	0.981
*All tobacco products are dangerous*	102/153 (66.7%)	20/47 (42.6%)	122/200 (61%)	**< 0.001**
*No discussion about electronic smoking during medical visits*	26/153 (17%)	14/47 (29.8%)	40/200 (20%)	0.055

**Table 8:** Comparison of variables associated with htps’ use

Variables	HTP Use No (%)	HTP Use Yes (%)	Total (%)	*p* value
*Gender (Male)*	88/164 (53.7%)	27/36 (47.2%)	105/200 (52.5%)	0.484
*Age > 14 years*	98/164 (59.8%)	30/36 (83.3%)	128/200 (64%)	**0.008**
*Influenced by peers or family*	10/164 (6.1%)	13/36 (36.1%)	23/200 (11.5%)	**< 0.001**
*Stress*	3/164 (1,8%)	5/36 (13,9%)	8/200 (4%)	**0.001**
*Sadness/apathy*	58/164 (35.4%)	15/36 (41.7%)	73/200 (36.5%)	0.477
*Relatives smoke*	76/164 (46.3%)	10/36 (27.8%)	86/200 (43%)	**0.042**
*Belief that electronic smoking is less harmful than traditional cigarettes*	142/164 (86.6%)	28/36 (77.8%)	170/200 (85%)	0.180
*All tobacco products are dangerous*	106/164 (64.6%)	16/36 (44.4%)	122/200 (61%)	**0.025**
*No discussion about electronic smoking during medical visits*	32/164 (17%)	8/36 (29.8%)	40/200 (20%)	0.713

## Discussion

To the best of our knowledge, apart from the National Tobacco Report [25], our is the first epidemiological study specifically addressing prevalence, attitudes, motivations, and risk awareness towards both e-cigarettes and heated tobacco products among the pediatric population in Italy. Our findings provide valuable insights into youth smoking behaviors (e.g. patterns of initiation, consumption habits), contributing to a more comprehensive understanding of the determinants influencing the uptake of such products. By assessing together the extent of use and its underlying motivations, our results offer an initial framework for developing targeted preventive strategies. Furthermore, our preliminary data represents a starting point for refining our survey thence expanding the sample size by involving additional research centers. A broader and more diverse cohort would enhance the generalizability of our findings, providing a robust basis for Public Health interventions aimed at reducing the appeal and consumption of e-cigarettes and HTPs among youth.

### E-cigarettes

A key finding emerging from our research is that a substantial 23% of participants declared active use of e-cigarettes. A closer examination reveals that albeit nearly half of ever-users reported only occasional use, suggesting experimentation, a substantial 40% vapes ten or more times per month, raising concerns about habitual use. This underscores the widespread presence of vaping among adolescents, aligning with global trends indicating that e-cigarette use is increasingly common among youth [25, 29, 30]. 

One of the significant associations explored is the relationship between e-cigarette use and age. Our data reveal that vapers tend to be older than non-users, since a significantly higher proportion of participants ≥ 14 years reported using e-cigarettes (89.4%), whereas non-users were predominantly younger (56.2%). While early adolescence represents a window of vulnerability, experimentation and regular use become more prevalent in mid-late adolescence. Reasonably, older adolescents are more exposed to environmental stimuli encouraging use (e.g. peer pressure, purchase autonomy). Early adolescence represents a crucial target for awareness campaigns, nonetheless preventive interventions should also embrace later teenage years, addressing the factors driving older adolescents toward vaping. The lower prevalence among youngest participants might reflect stricter parental and school regulations. However, this does not necessarily indicate reduced susceptibility; rather, it underlines the evolving nature of adolescent risk behaviors and the need for age-tailored prevention efforts [26, 27, 31]. 

Gender did not appear to influence e-cigarette use, as the distribution of users/non-users was similar between males and females. Available studies on gender differences in e-cigarette’s use are inconsistent [32]. Unlike traditional cigarette use, which has historically shown gender disparities, vaping behaviors are not confined to a specific sex group, reinforcing the need for prevention strategies targeting all adolescents rather than sex-specific interventions.

Device preference is a further significant consideration, with disposable e-cigarettes broadly favored (69%). Such preference raises concerns given the high nicotine content associated with disposable products. A small percentage (7%) is unaware of the type of device they use, which highlights a lack of awareness among the product and its detrimental effects on health. Additionally, the significant proportion of users consuming nicotine-containing e-cigarettes (91%) suggests a potential for dependence, particularly among those unaware of the nicotine content in their devices (13%). When stratified by age, the prevalence was significantly higher among users older than 14 years (97% vs. 81%) (*p* < 0.001). The widespread use of flavored e-cigarettes (76% of users) further underscores their appeal, with fruity-sweet (52%) and mentholated (15%) flavors being particularly popular. Our findings align with previous research demonstrating that flavoring enhances the attractiveness of smoking products, calling for stricter regulations on their marketing [33]. 

Nearly half of vapers have never purchased their e-cigarettes, implying that many obtain them through social sources, such as friends or relatives. The finding that 60% of underage users successfully purchased or even resold e-cigarettes despite legal restrictions highlights regulatory gaps. Moreover, the reported availability of e-cigarettes within schools is particularly alarming, implying that these environments may serve as informal distribution networks, further complicating prevention efforts [34]. 

The high prevalence of device-sharing is another noteworthy finding, with 73% of ever-users reporting occasional (69%) or habitual (4%) use of someone else’s device. Such majority indicates that youngsters share or borrow devices from friends or acquaintances, likely to integrate into social contexts or to try new flavors. This practice may contribute to increased experimentation and sustained use, particularly among non-owners [35]. 

Among non-users, 46% reported having no interest in trying e-cigarettes. However, a notable 23% indicated openness to smoking experimentation. Additionally, 16% stated they would probably not try vaping, though without complete certainty, while 15% expressed curiosity. These findings underscore again the persistent appeal of vaping among adolescents and the persistent need for implemented prevention strategies [4]. 

Efforts to quit demonstrate youngsters’ awareness of smoking-related harms, but reach varying levels of success, with many participants requiring multiple attempts. Almost 1 out of 5 users tried quitting 10 or more times, indicating significant efforts to overcome dependence, while 30% had never attempted to quit, possibly due to a lack of motivation or risk awareness. A concerning issue is the lack of external support. Among those attempting to quit, 70% relied solely on willpower, with minimal assistance from family or professionals.

### Heated tobacco products

Heated Tobacco Products represent an emerging area of engagement for adolescents. Among never-smokers, 76% expressed interest in trying these products, suggesting a growing curiosity towards new forms of tobacco consumption. Only 16% of participants reported current use. This percentage (in line with latest national data) [25] is relatively low but significant, as it suggests that a portion of the youth population has already begun experimenting. Notably, 2% of participants were uncertain about their usage, reflecting gaps in awareness. A significantly higher proportion of HTPs users were over the age of 14 (83.3%) compared to non-users (59.8%) (*p* < 0.05), implying that experimentation may be more common in mid-late adolescence. Gender distribution suggests a relatively balanced pattern. This aligns with previous research indicating that, unlike traditional cigarette smoking, HTPs’ use may not be strongly gender-dependent [32]. More than half reported only occasional use. However, a significant subset (34%) reported using HTPs ten or more times per month. These findings confirm a widespread pattern of occasional use alongside a noteworthy proportion of more regular users [25]. 

The notable proportion of users consuming nicotine-containing HTPs (34%) raises concerns about dependence. The strong preference for flavored HTPs (64%), with menthol (61%) and fruit flavors (29%) being the most favored, reinforces the key role of flavoring in product appeal [35]. 

Access to HTPs stands as a main regulatory challenge. While 37% of users reported never having purchased HTPs, 34% bought them from tobacco shops, 16% from vending machines, and 13% through third parties. Our results highlight the continued role of traditional points of sale and social networks in facilitating tobacco access among youths. Notably, among HTPs users obtaining products from third parties, 44% received them from underage providers. This challenges the effectiveness of current regulations and suggests the need for further and more consistent restrictions to protect young people from tobacco consumption [36]. The practice of using third-party products, reported by 56% of respondents, indicates that social access plays a crucial role in facilitating experimentation. Additionally, 20% admitted they would accept a product offered by a friend, reinforcing the powerful influence of peer dynamics on initiation.

Attempts to quit HTPs mirror e-cigs’ patterns, with 13% struggling with ten or more cessation attempts. Meanwhile, 26% had no intention of quitting, suggesting that a portion of users perceive HTPs as an acceptable long-term alternative to smoking. Similarly to vapers, only 30% sought external support. Adolescents attempting to quit both vaping and heated tobacco may face similar barriers, including a lack of accessible cessation resources and structured support systems.

### Psychosocial and environmental factors

Social influences emerge as the predominant factor driving e-cigarette use, with peer influence (35%) and curiosity (28%) reported by most users. These findings underline the role of social pressure in adolescent decision-making [26, 27, 31]. Among vapers, 47% reported that their attitudes toward smoking had been shaped by family or friends, compared to only 1% of non-users (*p* < 0.001). A significantly higher proportion of e-cigarette users (77%) reported having relatives who also smoke (any kind of cigarette) compared to non-users (51%) (*p* < 0.05). Interestingly, risk perception seems to play a minor role in shaping vaping behaviors, indicating that adolescents may underestimate e-cigarettes’ potential harms.

As far as HTPs are concerned, social influences emerge again as primary drivers of initiation, with peer influence (41%) and curiosity (31%) reported most frequently. Similarly to vapers, 36% of HTPs users recognized the impact of social influence, in contrast to 6% of non-users (*p* < 0.001). Moreover, the 72% of HTPs users reported having smokers in their household, compared to 54% of non-users (*p* < 0.05).

Growing up in an environment where smoking is normalized (or even encouraged) may reduce perceived risks and reinforce the idea that tobacco is socially acceptable. This emphasizes the importance of family-oriented interventions, encouraging smoke-free households and educating parents about their influence on children’s smoking choices. The significantly lower percentage of non-users acknowledging social influence could indicate a greater reliance on personal beliefs or parental guidance in shaping their attitudes toward smoking. Alternatively it is possible that users, having already engaged in consumption, retrospectively attribute their behavior to social factors as a way of rationalizing their choices. Therefore, is not clear whether social influence acts as a direct driver of initiation or simply reinforces pre-existing tendencies in subjects already predisposed to experimentation [4, 11, 37]. 

Our findings underline the importance of social dynamics, but adolescents’ vulnerability is further understood by exploring the role of social media and targeted marketing strategies. Tobacco-related content is widespread on social platforms (with up to half of youngsters reporting exposure) and such engagement significantly increases the likelihood of smoking initiation [38, 39]. Use of social media has been consistently associated with increased risk of e-cigarette use in a dose-response manner, emphasizing the need to regulate exposure and to restrict nicotine-related marketing on such platforms [40]. Additionally, exposure to social contents shared by peers and influencers reduces perceived risks, which highlights their pivotal role in shaping health behaviors [41]. From a different perspective, social media could be turned into an intervention channel, where prevention campaigns delivered by physicians and influencers may counteract industry-driven narratives. Future research with larger samples should therefore investigate the role of social media in shaping adolescent vulnerability.

### Awareness of smoking-related health effects

While Public Health efforts have succeeded in conveying the dangers of smoking to a large proportion of young people, misconceptions and underestimations persist: the scientific community advocates comprehensive campaigns addressing the risks of all nicotine products, including emerging alternatives like HTPs.

Our results point out a general lack of awareness about vaping’s detrimental health effects. The perception did not differ between vapers and non-users, while still considering e-cigarettes less harmful than traditional cigarettes These findings indicate a widespread normalization of electronic smoking devices, possibly fueled by marketing strategies emphasizing harm reduction, despite growing scientific evidence of their negative health consequences. Furthermore, we challenge the assumption that lower risk perception is the primary driver of e-cigarette use, suggesting instead that other factors - such as social acceptability or accessibility - may play a more significant role in influencing adolescent smoking behaviors [4, 27]. Interestingly, when asked to compare e-smoking with traditional cigarettes, 85% of participants considered electronic devices less harmful, while 12% believed the risks were comparable. Although such perception aligns with the proposed role of HTPs and e-cigarettes as harm reduction tools, it also raises concerns about possible unintended consequences. If the perceived risks is negligible, users may engage in more frequent consumption, counterweighing any reduction in harm.

Referring more generally to tobacco products-related health risks, danger awareness was by far more common among non-users than users. This significant difference suggests that underestimation of smoking effects may be susceptible to marketing claims portraying vaping and heated tobacco as “safer” alternatives to traditional smoking.

Moreover, misconceptions about nicotine content seem widespread, requiring improved education both for electronic smoking devices and HTPs These beliefs may influence smoking behaviors, as individuals misjudging nicotine’s presence may also underestimate its addictive potential. Clearer Public Health messaging (about nicotine content and its physiological effects) is decisive, as nicotine exposure plays a critical role in addiction potential and long-term health risks [42]. 

### Mental health and emotional well-being

Adolescents’ mental and emotional well-being was another key area of our concern. A troubling 37% of participants reported experiencing sadness, depression, or apathy. Although only 12% explicitly reported anxiety, these findings highlight a notable proportion of teenagers struggling with emotional distress. Given the recognized associations between negative mental health states and increased susceptibility to substance use, our results raise important questions about the potential psychological drivers behind smoking behaviors and reinforce the idea that addressing emotional well-being should be an integral part of tobacco prevention efforts [43, 44]. Feelings of sadness or apathy were more frequently reported among users than non-users. It is unclear whether vaping serves as a coping mechanism for emotional distress, or if individuals experiencing sadness are simply more likely to seek external stimuli, including nicotine use. Alternatively, this correlation could reflect different underlying psychological profiles between users and non-users, warranting further research to determine whether vaping exacerbates or alleviates emotional distress in youth. Contrarywise, the proportion of individuals reporting feelings of sadness was comparable among HTPs users and non-users, suggesting that heated tobacco consumption is not necessarily linked to emotional distress or self-medication behaviors. This contrasts with findings related to other tobacco and nicotine products, where emotional stress has been identified as a contributing factor [44]. Possibly, HTPs are adopted for reasons beyond emotional regulation, such as perceived convenience or social trends.

Stress also emerged as a significant variable, though with an intriguing pattern. Among vapers, 17% reported experiencing stress, whereas no non-users indicated feeling stressed. Such discrepancy suggests again two possible interpretations: either stress contributes to an increased likelihood of vaping, or else vapers perceive themselves as more stressed due to potential nicotine-related mood dysregulation. Nicotine’s short-term mood-altering effects might indeed provide temporary relief from stress, reinforcing habitual use [45]. However, this pattern could create a harmful cycle, where nicotine dependence exacerbates stress over time rather than alleviating it. The absence of reported stress among non-users could also reflect differences in stress-coping strategies. Further research is needed to clarify whether e-cigarettes serve as stress-relievers or if vapers experience higher stress levels due to external factors unrelated to nicotine consumption. Similarly, a significantly higher proportion of HTP users (14%) reported experiencing stress compared to only 2% of non-users.

### Prevention

The findings of our study also highlight significant gaps in the reach of anti-smoking awareness campaigns. Nearly half of participants reported no prior exposure to anti-smoking messaging. Current prevention efforts may not be sufficiently widespread or effectively targeted at adolescents. Strengthening the visibility and accessibility of prevention campaigns, particularly in schools and social media platforms, could enhance their impact.

Healthcare engagement regarding tobacco use appears insufficient, with a notable gap in communication. An overwhelming 80% of participants (with non-significant differences between users and non-users) reported never being asked about their smoking habits during medical visits. Interestingly, only 8% of children under 14 years (age of transition to high school) reported being asked about tobacco use, compared to 27% of older ones. Younger adolescents, particularly those in middle school, are systematically overlooked in smoking-related anamnesis. Lacking counseling and risk communication in routine healthcare constitutes a missed opportunity for prevention and early intervention. As recently advised by the Italian Pediatric Respiratory Society, strengthening the active role of healthcare providers in tobacco education could help address misinformation and encourage healthier choices among youth [46].

Some limitations of the present study should be acknowledged. First, the relatively small sample and the monocentric nature may limit the generalizability of our findings. However, these constraints are inherent to the preliminary nature of our investigation, which was primarily intended to provide exploratory data and shape the design of larger-scale, multicenter studies. The use of a self-reported questionnaire may be susceptible to response or recall bias. To mitigate this effect, we employed an anonymous digital survey administered in a non-supervised setting, thereby reducing external influences on participants’ answers. Our survey did not explore parental educational level and socioeconomic status, which may represent relevant predictors of adolescents’ tobacco use, other than the specific role of social media in shaping youngsters’ behaviors. Future multicenter studies ought to incorporate these variables to provide a more comprehensive picture. In our analysis, we employed descriptive statistics and chi-square tests, without multivariate analyses. Upcoming research with larger sample sizes should apply multivariate models to identify independent predictors of e-cigarettes and HTPs’ use. Studies involving a wider and more diverse cohort, and integrating objective measures where feasible, are warranted to strengthen the reliability and applicability of our results.

## Conclusions

The widespread normalization of electronic smoking devices among youths represents an ongoing Public Health concern. Despite existing regulations, underage access remains alarmingly frequent, demanding stricter enforcement strategies. The misperception of e-cigs and HTPs as safer alternatives to traditional smoking - largely fueled by aggressive misleading marketing and lacking risk communication - has led to concerning rates of experimentation and sustained use during a vulnerable stage of development. Furthermore, the association with mental issues warrants urgent consideration, as nicotine may exacerbate psychological distress, rather than serve as a coping mechanism. Given the evolving nature of tobacco delivery systems and the lack of independent long-term safety data, a multifaceted approach is required, combining adaptive and precautionary regulatory measures, enhanced community awareness campaigns, integration of mental health support, and active involvement of healthcare professionals. School-based interventions, digital awareness campaigns, and youth-centered outreach strategies should be implemented to ensure that adolescents receive accurate and compelling information about smoking-associated risks. Additionally, proactive screening in clinical settings and integration of tobacco prevention into routine healthcare could help identify at-risk individuals and provide targeted support. Future research should explore longitudinal patterns of use to better understand the trajectory of youth nicotine consumption and its long-term health implications.

## Supplementary Information

Supplementary Material 1.

## Data Availability

Data presented in this study are not publicly available but stored within the research group’s database at Sapienza University of Rome.

## Abbreviations

E-cigarettes or E-cigs: Electronic cigarettes
HTPs: Heated Tobacco Products
