# Supplementary material for: Prevalence, risk perception, and motivations behind E-cigarettes and heated tobacco use: a cross-sectional study in Italian adolescents
Source: Ital J Pediatr. 2025 Nov 17;51:303. doi: 10.1186/s13052-025-02144-y (PMC12625768; doi:10.1186/s13052-025-02144-y)
Supplement: Supplementary file 1 — Supplementary Material 1. [file 13052_2025_2144_MOESM1_ESM.docx]

**INSTRUCTION N°1**

The first few questions ask for some basic information about you.

**1. Everyone**

How old are you?

A. 11 years old

1. 12 years old
2. 13 years old
3. 14 years old
4. 15 years old
5. 16 years old
6. 17 years old
7. 18 years old

**2. Everyone**

Which is your gender?

1. Male
2. Female

**3. Everyone**

Which class do you attend?

1. I year of lower secondary school
2. II year of lower secondary school
3. III year of lower secondary school
4. I year of high school
5. II year of high school
6. III year of high school
7. IV year of high school
8. V year of high school

**INSTRUCTION N°2**

The following questions cover your use of particular types of tobacco products, such as e-cigarettes and heated tobacco products.

**INSTRUCTION N°3**

The next questions are about e-cigarettes or e-cigarettes, such as JUUL, SMOK, Suorin, Vuse, blue, Puff Bar, or STIG. You may also know them as vapes, mods, e-cigs, e-hookahs, or vape-pens. In this survey, these products and devices will be called e-cigarettes. E-cigarettes are battery-powered devices that usually contain a nicotine-based liquid that is vaporized and inhaled.

**4. Everyone**

Have you ever used an e-cigarette, even once or twice?

1. YES --> CONTINUE TO QUESTION 5
2. NO --> SKIP TO QUESTION 18

**5. E-cigarette users**

How old were you when you first used an e-cigarette?

1. 11 years old
2. 12 years old
3. 13 years old
4. 14 years old
5. 15 years old
6. 16 years old
7. 17 years old
8. 18 years old

**6. E-cigarette users**

In the last 30 days, how many days have you used e-cigarettes?

1. 1 time
2. 2 times
3. 3 to 5 times
4. 6 to 9 times
5. 10 or more times

**7. Current or past e-cigarette users**

Why did you use or do you currently use e-cigarettes? (Select one or more answers)

1. A friend uses/used them
2. A family member uses/used them
3. To stop using traditional cigarettes
4. They cost less than traditional cigarettes
5. They were easier to obtain than traditional cigarettes
6. I've seen people on TV, online, or in movies using them
7. They are less harmful than traditional cigarettes
8. They were available in flavors such as menthol, mint, candy, fruit, or chocolate
9. I can use them unnoticed at home or school
10. I was curious about
11. I felt anxious, stressed, or depressed
12. Other reasons

**8. E-cigarette users in the last 30 days**

Which of the following best describes the type of e-cigarette you have used in the last 30 days? If you've used more than one type, think about the one you use most often.

1. A disposable e-cigarette (e.g. Puff Bar or STIG)
2. An e-cigarette that uses pre-filled or refillable capsules or cartridges (e.g., JUUL, SMOK)
3. An electronic cigarette with a tank refillable with liquids (including mod systems that can be customized by the user)
4. I don't know the type

**9. E-cigarette users in the last 30 days**

Did some of the e-cigarettes you have used in the last 30 days contain nicotine?

1. Yes
2. No
3. I don't know

**10. E-cigarette users in the last 30 days**

Were some of the e-cigarettes you've used in the last 30 days flavored?

1. Yes
2. No
3. I don't know

**11. Users of flavored e-cigarettes in the last 30 days**

What flavor were the e-cigarettes you used in the last 30 days? (Select one or more)

1. Menthol
2. Mint
3. Cloves or spices
4. Fruit
5. Chocolate
6. Alcoholic beverages (such as wine, margaritas, or other cocktails)
7. Candies, desserts, or other sweets
8. Some other flavor not listed here

**12. E-cigarette users who have purchased the products by themselves**

In the last 30 days, where have you purchased e-cigarette devices, capsules, cartridges, or e-liquid refills? (Select one or more)

1. I haven't purchased e-cigarettes in the last 30 days
2. I bought them from another person (a friend, family member, or someone else)
3. A gas station or convenience store
4. A vending machine
5. A vape shop or tobacco shop
6. Some other places not listed here

**13. E-cigarette users who have received them from someone else**

Previously, you claimed to have received or purchased e-cigarette devices, capsules, cartridges, or e-liquid refills from another person, such as a friend or family member, in the last 30 days. How old was this person?

1. Under 18 years old
2. Over 18 years old

**14. E-cigarette users**

Have you ever obtained or purchased an e-cigarette device (including disposables), a pod, cartridge, single puff, or e-liquid refill while at school or school property?

1. Yes
2. No

**15. E-cigarette users in the last 30 days**

In the last 30 days, how often have you used someone else's e-cigarette device?

1. Never
2. Sometimes
3. Most of the time
4. Always

**16. E-cigarette users in the last 30 days**

In the last 12 months, how many times have you stopped using e-cigarettes for a day or more because you were trying to stop definitively using e-cigarettes?

1. I haven't tried to quit in the last 12 months
2. 1 time
3. 2 times
4. 3 to 5 times
5. 6 to 9 times
6. 10 or more times

**17. E-cigarette users who have reported ≥ 1 attempt to quit in the past 12 months**

When you tried to stop using e-cigarettes, did you use any of the following? (Select one or more)

1. I didn't use any resources
2. Help or advice from a parent or guardian
3. Help or advice from a friend or schoolmate
4. Help or advice from a teacher or coach
5. Help or advice from a doctor or healthcare professional
6. Help or advice found on the internet
7. A mobile app or messaging program
8. A helpline
9. Something else

**18. Subjects who have never been e-cigarette users**

Have you ever been curious to try an e-cigarette?

1. Definitely yes
2. Probably yes
3. Probably not
4. Surely not

**19. Subjects who have never been e-cigarette users**

If one of your best friends offered you an e-cigarette, would you use it?

1. Definitely yes
2. Probably yes
3. Probably not
4. Surely not

**INSTRUCTION N°4**

The next section is about "heated tobacco products" such as iQOS, glo, and Eclipse. Heated tobacco products heat tobacco sticks or capsules to produce a vapor. They are different from e-cigarettes, which heat a liquid to produce a vapor. DON'T THINK ABOUT E-CIGARETTES WHEN ANSWERING THE FOLLOWING QUESTIONS.

**20. Everyone**

Have you ever used a "heated tobacco product", even just once or twice?

1. Yes --> CONTINUE TO QUESTION 21
2. No --> SKIP TO QUESTION 34
3. I don't know/I'm not sure --> CONTINUE TO QUESTION 21

**21. Heated tobacco product users**

How old were you when you first used an heated tobacco product?

1. 11 years old
2. 12 years old
3. 13 years old
4. 14 years old
5. 15 years old
6. 16 years old
7. 17 years old
8. 18 years old

**22. Heated tobacco product users or unsure**

In the last 30 days, how many days have you used a "heated tobacco product"?

1. 1 time
2. 2 times
3. 3 to 5 times
4. 6 to 9 times
5. 10 or more times

**23. Current or past heated tobacco product users**

Why did you use or do you currently use heated tobacco products? (Select one or more answers)

1. A friend uses/used them
2. A family member uses/used them
3. To stop using traditional cigarettes
4. They cost less than traditional cigarettes
5. They were easier to obtain than traditional cigarettes
6. I've seen people on TV, online, or in movies use them
7. They are less harmful than traditional cigarettes
8. They were available in flavors such as menthol, mint, candy, fruit, or chocolate
9. I can use them unnoticed at home or school
10. I was curious about
11. I felt anxious, stressed, or depressed
12. Other reasons

**24. Heated tobacco product users in the last 30 days**

Did some of the heated tobacco products you have used in the last 30 days contain nicotine?

1. Yes
2. No
3. I don't know

**25. Heated tobacco products users in the last 30 days**

Any of the heated tobacco products you've used in the last 30 days was flavored?

1. Yes
2. No
3. I don't know

**26. Consumers of flavoured heated tobacco products in the last 30 days**

What flavors were the heated tobacco products you used in the last 30 days? (Select one or more answers)

1. Menthol
2. Mint
3. Fruit
4. Candies, desserts, or other sweets
5. Some other flavor not listed here

**27. Consumers of heated tobacco products who have purchased the products by themselves**

In the last 30 days, where have you bought your heated tobacco products? (Select one or more answers)

1. I haven't bought any heated tobacco products in the last 30 days
2. I bought them from another person (a friend, family member, or someone else)
3. A gas station or convenience store

D. A vending machine

1. A vape shop or tobacco shop
2. Some other places not listed here

**28. Consumers of heated tobacco products who have received them from someone else**

Previously, you claimed to have received or purchased your heated tobacco products from another person, such as a friend or family member in the last 30 days. How old was this person?

1. Under 18 years old
2. Over 18 years old

2**9. Users of heated tobacco products in the last 30 days**

In the last 30 days, has anyone refused to sell you heated tobacco products because of your age?

At. I haven't tried to buy heated tobacco products in the last 30 days

B. Yes

C. No

**30. Users of heated tobacco products**

How easy do you think it is for people your age to buy heated tobacco products in a store?

1. Easy
2. Pretty easy
3. Not easy at all
4. Difficult

**31. Heated tobacco users in the last 30 days**

In the last 30 days, how often have you used someone else's heated tobacco device?

1. Never
2. Rarely
3. Sometimes
4. Most of the time
5. Always

**32. Users of heated tobacco products in the last 30 days**

In the last 12 months, how many times have you stopped using heated tobacco products for a day or more because you were trying to definitively using heated tobacco products?

1. I haven't tried to quit in the last 12 months
2. 1 time
3. 2 times
4. 3 to 5 times
5. 6 to 9 times
6. 10 or more times

**33. Heated tobacco product users who have reported ≥ 1 attempt to quit in the past 12 months**

When you tried to stop using heated tobacco products, did you use any of the following? (Select one or more)

1. I didn't use any resources
2. Help or advice from a parent or guardian
3. Help or advice from a friend or schoolmate
4. Help or advice from a teacher or coach
5. Help or advice from a doctor or healthcare professional
6. Help or advice found on the internet
7. A mobile app or messaging program
8. A helpline
9. Something else

**34. Subjects who have never been heated tobacco product users**

Have you ever been curious to try a heated tobacco device?

1. Definitely yes
2. Probably yes
3. Probably not
4. Surely not

**35. Subjects who have never been heated tobacco product users**

If one of your best friends offered you a heated tobacco product, would you use it?

1. Definitely yes
2. Probably yes
3. Probably not
4. Surely not

**36. Everyone**

Have you been seen by a doctor or healthcare professional in the last 12 months?

1. Yes --> CONTINUE TO QUESTION 37
2. No --> SKIP TO QUESTION 38

**37. Respondents who have undergone a medical visit in the last 12 months**

During any of these visits to a doctor, dentist, nurse or other healthcare professional, were you asked if you used tobacco products (of any kind: traditional cigarettes, e-cigarettes, IQOS)?

1. Yes
2. No

**38. Everyone**

In the last 12 months, have you seen or heard anti-tobacco ads on television, the internet, social media, or on the radio?

1. Yes
2. No

**39. Everyone**

How much do you agree with the statement "all tobacco products are dangerous"?

1. Fully agree
2. Somewhat agree
3. Disagree
4. Strongly disagree

**40. Everyone**

How much do you think people get harmed by smoking e-cigarettes?

1. No damage
2. Minimal damage
3. Significant damage

**41. Everyone**

Electronic cigarettes:

1. Never contain nicotine
2. Sometimes contain nicotine
3. Usually contain nicotine
4. Always contain nicotine

**42. Everyone**

Do you believe that e-cigarettes create, compared to traditional cigarettes:

1. Less addiction
2. The same addiction
3. More addiction
4. I don't know enough about

**43. Everyone**

How much do you think people get harmed by smoking heated tobacco products?

1. No damage
2. Minimal damage
3. Significant damage

**44. Everyone**

Heated tobacco products:

1. Never contain nicotine
2. Sometimes contain nicotine
3. Usually contain nicotine
4. Always contain nicotine

**45. Everyone**

Do you believe that Heated tobacco products create, compared to traditional cigarettes:

1. Less addiction
2. The same addiction
3. More addiction
4. I don't know enough about

**46. Everyone**

Compared to traditional cigarettes, e-cigarettes and heated tobacco products (e.g. IQOS) cause:

1. Less damage
2. The same damage
3. More damage
4. I don't know

**47. Everyone**

In the last 30 days, how many days have you been exposed to smoke from someone smoking (any type of cigarette) in an outdoor public place? Examples of outdoor public places are schoolyards, sidewalks, parking lots, stadiums, and parks.

1. 0 days
2. 1 or 2 days
3. 3 to 5 days
4. 6 to 9 days
5. 10 to 19 days
6. 20 to 29 days
7. All 30 days

**48. Everyone**

In the last 30 days, how many days have you been exposed to the vapor of someone smoking (any type of cigarette) in an indoor public place? Examples of indoor public places are school buildings, shops, restaurants and sports arenas. Don't think about houses when answering this question.

1. 0 days
2. 1 or 2 days
3. 3 to 5 days
4. 6 to 9 days
5. 10 to 19 days
6. 20 to 29 days
7. All 30 days

**49. Everyone**

Someone who lives with you now... (Select one or more answers)

1. Uses e-cigarettes
2. Smokes traditional cigarettes
3. Smokes cigars
4. Uses chewing tobacco
5. Smokes rolled cigarettes
6. Uses soluble tobacco products
7. Uses heated tobacco products
8. No one who lives with me now uses any form of tobacco

**50. Everyone**

Which, of the following statements, best reflects your mood over the past two weeks?

1. I found low interest in doing things
2. I felt down, depressed, or hopeless
3. Not being able to control or stop worries, I felt anxious
4. I felt happy
5. I felt calm
